# A Multifrequency Brain Network-Based Deep Learning Framework for Motor Imagery Decoding

**DOI:** 10.1155/2020/8863223

**Published:** 2020-12-07

**Authors:** Juntao Xue, Feiyue Ren, Xinlin Sun, Miaomiao Yin, Jialing Wu, Chao Ma, Zhongke Gao

**Affiliations:** ^1^School of Electrical and Information Engineering, Tianjin University, Tianjin 300072, China; ^2^Department of Neurorehabilitation and Neurology, Tianjin Huanhu Hospital, Tianjin Key Laboratory of Cerebral Vascular and Neurodegenerative Diseases, Tianjin Neurosurgical Institute, Tianjin 300350, China

## Abstract

Motor imagery (MI) is an important part of brain-computer interface (BCI) research, which could decode the subject's intention and help remodel the neural system of stroke patients. Therefore, accurate decoding of electroencephalography- (EEG-) based motion imagination has received a lot of attention, especially in the research of rehabilitation training. We propose a novel multifrequency brain network-based deep learning framework for motor imagery decoding. Firstly, a multifrequency brain network is constructed from the multichannel MI-related EEG signals, and each layer corresponds to a specific brain frequency band. The structure of the multifrequency brain network matches the activity profile of the brain properly, which combines the information of channel and multifrequency. The filter bank common spatial pattern (FBCSP) algorithm filters the MI-based EEG signals in the spatial domain to extract features. Further, a multilayer convolutional network model is designed to distinguish different MI tasks accurately, which allows extracting and exploiting the topology in the multifrequency brain network. We use the public BCI competition IV dataset 2a and the public BCI competition III dataset IIIa to evaluate our framework and get state-of-the-art results in the first dataset, i.e., the average accuracy is 83.83% and the value of kappa is 0.784 for the BCI competition IV dataset 2a, and the accuracy is 89.45% and the value of kappa is 0.859 for the BCI competition III dataset IIIa. All these results demonstrate that our framework can classify different MI tasks from multichannel EEG signals effectively and show great potential in the study of remodelling the neural system of stroke patients.

## 1. Introduction

Stroke is a common brain disease and becomes the third most common cause of death [1]. It could cause brain nerve cell damage or necrosis, leading to limb discordance, spasm, and even hemiplegia [2]. Since standard physical therapy is costly and limited [3], there has been a lack of effective stroke treatment. The functional rehabilitation of stroke patients mainly depends on neural plasticity [4]. The motor imagery (MI) paradigm is simple and inexpensive [5]. It could transform the subject's motor intention into control signals independent from normal nerves and muscles. Existing findings [6–8] have indicated that most stroke patients retain MI nervous feedback function even if other neurological functions are affected. Based on the above factors, the MI paradigm-based rehabilitation system has received extensive attention [9, 10].

Brain-computer interface (BCI) can convert the intention of moving in the brain of stroke patients into control signals to control muscles and nerves. Therefore, decoding the patient's movement intention accurately by EEG has a significant impact on the rehabilitation of the system's performance. However, the raw MI signals usually contain much noise and are highly nonlinear. Those factors pose a great challenge for decoding MI-based EEG signals effectively. Researchers have proposed numerous algorithms to process MI signals [11, 12], including wavelet transform model [13], empirical mode decomposition [14], and common spatial pattern (CSP) [15]. Among them, CSP is the most popular method to extract features associated with different MI tasks [16, 17]. The improved method based on traditional CSP has also achieved impressive results. Jin et al. [18, 19] proposed an RCSP method-based correlation channel selection method, and they advised a theory-based Dempster-Shafer algorithm to improve the feature selection of the objective function to reduce the time consumption of the CSP algorithm. The filter bank CSP (FBCSP) algorithm has been widely used in recent years [20]; it improves the CSP algorithm's frequency-sensitive features. The FBCSP algorithm selects the appropriate frequency bands automatically; therefore, it can obtain better results on different subjects [21]. Previous works [22–25] have proved that FBCSP shows better performance and adaptability than the classical CSP algorithm. Furthermore, FBCSP could be extended to a one-versus-rest filter bank common spatial pattern (OVR-FBCSP) algorithm for multiclass problems.

The brain can be considered a dynamic network, which consists of numerous neurons. In recent years, the complex network that derives from complex systems has been proven to be a practical approach in brain state research [26, 27]. Specifically, one can set the electrodes of the brain as the nodes, and the edges can be estimated via diverse correlation measure algorithms, such as phase lag index, Spearman rank correlation, and phase locking value (PLV). Up to now, various brain network methods have been utilized to analyse EEG signals. Li et al. [28] constructed a P300-based brain network via the coherence between electrodes and compared the differences under different stimulation conditions. Yang and Gao [29] proposed a multivariate weighted ordinal pattern transition (MWOPT) network to analyse the driving fatigue behaviour and obtain high accuracy. Ai et al. [30] constructed a single-layer brain network by canonical correlation analysis and combined CSP and local characteristic-scale decomposition to extract the feature of MI signals. Besides, the multifrequency network, as the single-layer network development, can analyse the system from different perspectives. Thus, the characteristics of the system can be expressed more comprehensively.

Compared with classical machine learning, deep learning has a mightier ability to characterize complex systems. In fact, many researchers have applied deep learning to the BCI system and achieved many remarkable achievements [31, 32]. Gao et al. [33] proposed a method combined integrating complex network and broad learning system to study visual evoked potential, and the results showed that deep learning demonstrated better performance than traditional methods. Leon et al. [34] proposed a network based on artificial neural networks and recurrent neural networks to identify MI-based signals automatically. Chen et al. [35] proposed a decoding method based on filter-space and time-space convolution, which shows significant performance improvement on two datasets. As a typical representative of deep learning, the convolutional neural network (CNN) has made remarkable achievements in computer vision [36], malware-detection [37], and many other fields. Applying CNN to MI-based BCI systems for classification has become a hot topic. For example, Gao et al. [38] developed an EEG-based spatial-temporal CNN network to extract temporal dependencies from EEG signals and got 97.37% classification accuracy in driver fatigue dataset. Zhang et al. [39] proposed a novel method based on CNN long short-term memory (LSTM) and achieved satisfactory results in multiclass MI problems. Reviewing the overview of complex networks and deep learning [40], the previous work usually only focuses on the time or frequency domain of MI signals and does not take full advantage of the deep learning in describing multiple types of characteristics. The fusion of these information contributes to building an efficient MI-based BCI system.

**Figure 1:**
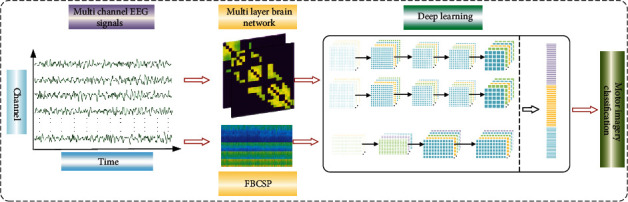
The framework of our model.

Traditional metric parameters for complex networks, such as aggregation coefficient and average path length, are often limited to a single view of the brain network and do not fully exploit the richness information hidden in the topology. Deep learning can automatically extract abstract features from the input and describe complex relationships. Combining brain networks and deep learning can preserve the information extracted from brain networks to the greatest extent possible. Motivated by the described background and challenges, we propose a novel deep learning framework based on the multifrequency brain network for motor imagery decoding. Specifically, multichannel MI signals are divided into two frequency bands. Then, a multilayer brain network where edges are determined via PLV is derived. Each layer corresponds to a frequency band. The multilayer brain network considers both the frequency characteristics and the interchannel coupling relationship of the multichannel EEG signals. Next, a multiple frequency convolutional neural network (MFCNN) framework is designed for the brain network, taking the multilayer brain networks as input. Meanwhile, we design a model with a special convolutional kernel that allows it to learn the reorganized features efficiently, which was obtained by the FBCSP algorithm. The outputs of the two models are concatenated together for classification. To evaluate the performance of the framework, we verify our framework on public BCI competition IV dataset 2a and public BCI competition III dataset IIIa and obtain impressive results in both datasets. Specifically, the accuracy of the first dataset is 83.38%, and the value of kappa is 0.784; the accuracy of the second dataset is 89.45%, and the value of kappa is 0.859. The proposed framework combines the multiscaled features and CNN-based deep learning networks, which could improve the classification accuracy effectively in different subjects. The framework of our model is shown in Figure 1.

## 2. Materials

### 2.1. Dataset I: The Public BCI Competition IV Dataset 2a

**Figure 2:**
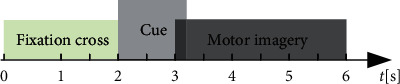
Time scheme of one session in BCI competition IV dataset 2a.

The public BCI competition IV dataset 2a is used to study the multicategory MI tasks. It contains four categories, including the right hand, left hand, foot, and tongue. The dataset contains EEG signals from nine healthy subjects, and they are A01, A02,…, A09. Each subject includes 288 train and test sessions. The data is sampled at 250 Hz and includes 22 EEG channels and three monopolar electrooculogram (EOG) channels. It is processed with 0.5 Hz to 100 Hz bandpass filter, and a notch filter of 50 Hz eliminates power frequency interference. The time scheme of one single trial is shown in Figure 2. At the beginning of each trial (*t* = 0 s), a fixation cross appears on the screen. After 2 seconds (*t* = 2 s), a cue will display in the form of arrows, which corresponds to four classes. It instructs the subject to begin the motor imagery task and maintains 3 s until the end of trial (*t* = 6 s). More detailed dataset description can be found in [41].

### 2.2. Dataset II: The Public BCI Competition III Dataset IIIa

**Figure 3:**
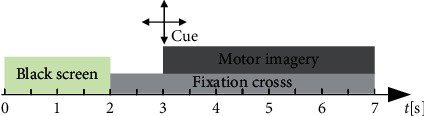
Time scheme of one session in BCI competition III dataset IIIa.

The public BCI Competition III dataset IIIa is used here to validate the reliability of the method. Four kinds of imagery movements are considered in this dataset, including the right hand, left hand, foot, and tongue. The raw data are recorded on 60 channels at a sampling frequency of 250 Hz. Three subjects participated in the experiment, and they are “k3b,” “k6b,” and “l1b.” The time scheme of one single trial is shown in Figure 3. A blank screen is displayed for the first two seconds (*t* = 0 s) of each trial, and a fixed cross is displayed for the next one second (*t* = 2 s). At *t* = 3 s, an arrow will point left, right, up, and down, which prompts the subject to imagine movements of the right hand, left hand, foot, and tongue, and this process will continue for 4 s until *t* = 7 s. The numbers of trials are 360, 240, and 240 for “k3b,” “k6b,” and “l1b,” respectively.

## 3. Methodology

**Figure 4:**
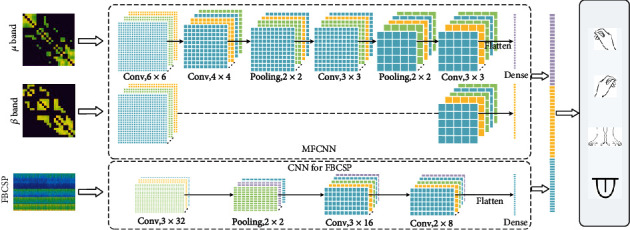
The architecture of the deep learning model. Conv, 6 × 6 indicates the convolution kernel size is 6 × 6.

In this part, we propose a novel deep learning framework based on a multifrequency brain network, which could take full advantage of the brain network and deep learning. Firstly, we construct a multifrequency brain network (MFCNN) in the *μ* band and *β* band and design a multiple frequency convolutional neural network to extract features. Further, in order to extract more precise frequency band information, we introduce 43 frequency bands and the FBCSP algorithm. A CNN-based deep learning framework is designed to learn the features of the FBCSP algorithm. Finally, all outputs are concatenated for classifying MI tasks with a softmax function. The detailed structure of the entire framework is provided in Figure 4. Next, we will introduce them as follows.

### 3.1. Multifrequency Brain Network Construction

Multichannel MI signals reflect the activity directly from different brain regions, which present a significant frequency dependence. When subjects perform different MI tasks, different motor sensation cortexes of the brain would be activated, and specific physiological phenomena such as event-related synchronization (ERS) and event-related desynchronization (ERD) would appear. In MI tasks, the *μ* band (8–12 Hz) and *β* band (18–24 Hz) are the major spectrum of ERS and ERD. We design two filters to filter the raw MI signals in the *μ* and *β* bands. For the *μ* band (*β* band is similar), we set the electrodes as nodes, and the PLV algorithm derives the edges between the nodes.

For the MI signals *x*(*t*) and *y*(*t*) from two channels, the instantaneous phase should be calculated first. The analytic of *x*(*t*) can be expressed by the following formula:

(1) $$\begin{array}{c} Z_{x}\left(t\right)=x\left(t\right)+i\tilde{x}\left(t\right)=A_{x}\left(t\right)e^{\varphi_{x}\left(t\right)}, \end{array}$$

where $\tilde{x}\left(t\right)$, *A*_*x*_(*t*), and *φ*_*x*_(*t*) are the imaginary part, amplitude, and instantaneous phase of*Z*_*x*_(*t*), respectively. $\tilde{x}\left(t\right)$ is obtained by the Hilbert transformation:

(2) $$\begin{array}{c} \tilde{x}\left(t\right)=\frac{1}{\pi }p.v\int_{-\infty }^{+\infty }\frac{x\left(a\right)}{x\left(t-a\right)}da, \end{array}$$

where *p*.*v* is Cauchy principal value. *A*_*x*_(*t*) and *φ*_*x*_(*t*) can be calculated by

(3) $$\begin{array}{c} \left\{\begin{array}{c} A_{x}\left(t\right)=\sqrt{\left(x^{2}\left(t\right)+i{\tilde{x}}^{2}\left(t\right)\right)},\\ \varphi_{x}\left(t\right)=\arctan\frac{\tilde{x}\left(t\right)}{x\left(t\right)}. \end{array}\right. \end{array}$$

For the MI signals *y*(*t*) of another channel, the corresponding instantaneous phase *φ*_*y*_(*t*) can be obtained by a similar step. Finally, the PLV is calculated by quantifying the instantaneous phase difference *φ*_*xy*_(*t*) = *φ*_*x*_(*t*) − *φ*_*y*_(*t*):

(4) $$\begin{array}{c} {\text{PLV}}_{x,y}=\left|{\langle e^{\varphi_{xy}\left(t\right)}\rangle }_{t}\right|=\sqrt{{\langle \cos\varphi_{xy}\left(t\right)\rangle }_{t}^{2}+{\langle \sin\varphi_{xy}\left(t\right)\rangle }_{t}^{2}}. \end{array}$$

<·>_*t*_ stands for the mean in the *t* time range, and the range of PLV is 0-1. The PLV indicates the degree of synchronization between *x*(*t*) and *y*(*t*); according to the above formula, the value of PLV is only related to the phase of MI signals and is not influenced by the amplitude of signals. In addition, it can respond to the phase information of the signal in the narrow band frequency range (*μ* and *β* bands) intuitively, and this information reflects the physiological mechanism of the brain activity.

Mathematically, the network calculated by Equation (4) is a correlation matrix. In order to get brain networks, 75% of the weak links in the matrix are discarded. We derive brain networks in the two frequency bands separately and obtain a multifrequency brain network with two layers (corresponding to the *μ* and *β* bands, respectively).

### 3.2. Multiple Frequency Convolutional Neural Network

We take a multifrequency brain network as input, and a multiple frequency convolutional neural network (MFCNN) is designed to classify MI signals. Each layer of the MFCNN corresponds to a frequency band of the multifrequency brain network. The convolutional layers are invariant to local transitions and invariant to location, so this structure could learn and integrate the rich topology hidden in rhe multilayer brain network effectively. We design the same network structure for different brain network layers, and their outputs are concatenated together. More MFCNN model parameters are presented in Table 1.

Next, we will take the first layer (corresponding to the *μ* band) of MFCNN as an example to illustrate the detail of the deep learning network. Specifically, each layer of the MFCNN model consists of two blocks. Multiple convolutional and pooling layers serve as the core of the first block.

(5) $$\begin{array}{c} {\left(O_{i,j}\right)}_{n}^{p}=f\left(\text{conv}\left(w_{n},I_{i,j}\right)+b_{n}\right), \end{array}$$

where *I*_*i*,*j*_ denotes the input block centered at the position (*i*, *j*). The *p*th convolutional layer contains *n* convolutional kernels; *W*_*k*_ and *b*_*k*_ correspond the weight matrix and the bias; and *f*(·) is the activation function. Each convolution kernel uses exponential linear units (ELU) [42] as the activation function. The ELU function has the following form:

(6) $$\begin{array}{c} g\left(x\right)=\left\{\begin{array}{c} x,\;x>0,\\ \lambda \left(e^{x}-1\right),\;\text{otherwise}. \end{array}\right. \end{array}$$

*λ* is a parameter that can be adjusted by the back-propagation algorithm automatically. After the convolutional layer, the maxpooling layer is added. The maxpooling layer achieves downsampling from the perspective of shift invariance; it reduces parameters while maintaining the main features, prevents overfitting, and improves the generalization of the model. Maxpooling function can be realized via the following:

(7) $$\begin{array}{c} \left(O_{i,j}\right)=\max\text{pool}\left(\left(I_{p,q}\right)\right)\;\forall \left(p,q\right)\in X_{i,j}, \end{array}$$

where *X*_*i*,*j*_ denotes the adjacent region around the position (*i*, *j*). maxpool(·) means to select the biggest parameter from the matrix. In the first block, the kernel size of the maxpooling layer is 2 × 2.

The second block is used to extract more high-level features, which contain two convolutional layers and a maxpooling layer. Both convolutional layers use the same structure, consist of 64 convolutional kernels, the size of kernel is 3 × 3, and take the ELU as the activation function. The size of maxpooling kernel is 2 × 2.

After the two blocks, the flatten layer is used to expand the data from convolution layer into one dimension. Finally, a fully connected layer (dense layer) is added to integrate the features.

### 3.3. The Filter Bank Common Spatial Pattern

**Figure 5:**
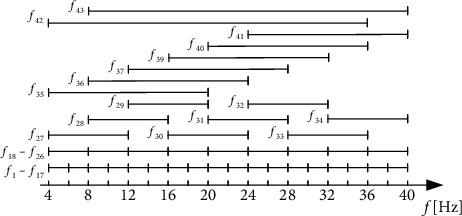
The band range of the filter banks.

Before the OVR-FBCSP algorithm, a filter bank will be employed to decompose the MI signals into multiple frequency bands. The filter bank set consists of Chebyshev Type II filters and includes 43 frequency bands. The 43 bands' range covers 4-40 Hz, and they can cover the maximum amount of valid information.  Figure 5 shows the specific distribution of the frequency bands.

Next, we introduce 11 time windows to segment the raw MI signal, which greatly increase the number of features. The start time and end time of the time windows are shown in Table 2.

A four-class filter can be generated by combining four two-class filters [43]. The spatial filtering is obtained by linear transformation of MI signal with OVR-FBCSP algorithm as follows:

(8) $$\begin{array}{c} F_{f,i}=W_{f}^{T}X_{f,i}^{j}, \end{array}$$

where *X*_*f*,*i*_^*j*^ denotes the MI signals which is recorded in the *i*th trail, processed by the *f*th bandpass filter, and split by the *j*th time window. The size of *X*_*f*,*i*_^*j*^ is *N* × *M*, where *N* is the number of channels and *M* is the number of samples per channel. *F*_*f*,*i*_ denotes the feature matrix after spatial filtering. *W*_*f*_ denotes the projection matrix, which is calculated by FBCSP algorithm. And *T* denotes the transposition operation of the matrix. *W*_*f*_ can be expressed by the following formula:

(9) $$\begin{array}{c} W_{f}=\left[W_{f,1},W_{f,2},\cdots ,W_{f,n}\right], \end{array}$$

where [*W*_*f*,1_, *W*_*f*,2_, ⋯*W*_*f*,*n*_] denotes the weight of spatial filter. The matrix *W*_*f*,*n*_ denotes a spatial filter of one class versus others, where *n* denotes the number of MI tasks. It is proved by previous research [44, 45] that the solution of *W*_*f*,*n*_ can be transformed into the eigenvalue decomposition problem, as follows:

(10) $$\begin{array}{c} P_{f,k}W_{f,k}=\left(\overset{n}{\underset{k=1}{\sum }}P_{f,k}\right)W_{f,k}X_{f,k}, \end{array}$$

where *P*_*f*,*k*_ is the covariance matrices of the *k* class MI signals after filtering by the *f*th filter. The *X*_*f*,*k*_ denotes the diagonal matrix corresponding to *P*_*f*,*k*_ eigenvalues. The two pairs of CSP features of the *i*th trail for *f*th bandpass filtered MI signals are given by the following formula:

(11) $$\begin{array}{c} S_{f,i}=\mathrm{lg}\frac{\mathrm{diag}\left({\tilde{W}}_{f}^{T}X_{f,i}^{j}X_{f,i}^{jT}{\tilde{W}}_{f}\right)}{\text{tr}\left({\tilde{W}}_{f}^{T}X_{f,i}^{j}X_{f,i}^{jT}{\tilde{W}}_{f}\right)}, \end{array}$$

where ${\tilde{W}}_{f}$ is expressed as a matrix which consists of the first two columns and the last two columns from *W*_*f*_, where diag(·) returns the diagonal elements of matrix. *S*_*f*,*i*_ is the output of FBCSP, and tr(·) returns the trace of matrix. After that, *S*_*f*,*i*_ is reorganized into a two-dimension matrix. The vertical axis is arranged according to 43 frequency bands, and the horizontal axis is the FBCSP feature of each frequency band.

### 3.4. CNN-Based Deep Learning Framework for FBCSP

According to the characteristics of the FBCSP feature matrix, a special convolution kernel is designed to learn the feature of matrix. Through this structure, each convolution kernel can fuse the FBCSP features of the adjacent frequency bands.

Specifically, the size of convolutional kernel is *P* × *Q*. *P* that determines *P* neighbouring bands can be fused simultaneously in a convolutional operation, and a sequence of length *Q* will be learned. Compared with MFCNN networks, this framework will further refine the optimal band information. More detailed network structure is shown in Table 1.

The first convolutional layer of the network is used to extract the shallow features of the feature matrix, which consists of 32 convolutional kernels with a size of 3 × 32. It determines that three adjacent frequency bands could be convolved simultaneously. Then, a maxpooling layer is added to reduce redundant information (feature dimension), and the size of maxpooling layer kernel is 2 × 2. After that, two additional convolutional layers are added to extract deeper information, including 64 convolutional kernels with a size of 3 × 16 and 64 convolutional kernels with a size of 2 × 8. Finally, all features are flattened into one dimension, and the fully connected layer is added to integrate these features.

## 4. Results and Discussion

### 4.1. The Motor Imagery Characterization Based on Multifrequency Brain Network

**Figure 6:**
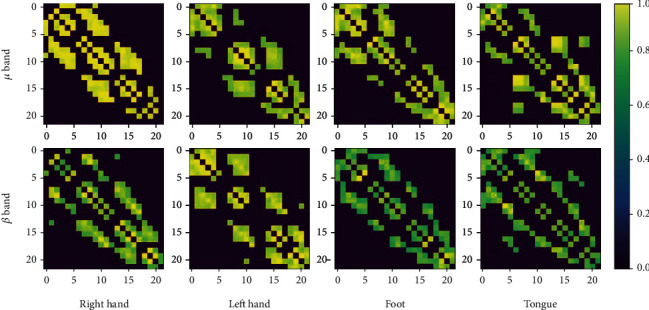
The multifrequency brain network constructed from different MI tasks.

The multifrequency brain network integrates channel-, time-, and frequency-related information, which could describe the state of different brain regions during MI tasks. We take subject A01 from dataset I as an example for visualization to demonstrate the brain activity during different MI tasks. In Figure 6, two multilayer brain networks are selected randomly and displayed. Each row corresponds to one specific frequency band of the multifrequency brain network, four columns correspond to different classes of MI tasks, and the number in each brain network corresponds to the electrode.

For brain networks with a specific frequency, the topology of different class brain networks shows a significant difference, and different frequency brain network topologies of the same class are very similar. The region of the tongue MI task network seems to cover a wider region and has a weaker connection than other tasks, because the tongue task would call more brain area compared with other tasks. Ai et al. [30] find tongue task calls more neurons, which strongly supports our findings. According to the electrode position in Figure 6, the connection of right-hand MI task is concentrated in the 7th-12th channels significantly, which corresponds to the area of the brain activity. This finding can be seen as an extension of the previous work [46].

### 4.2. Deep Learning Model for Classification

Based on the dataset described, 60% of samples are selected randomly as training dataset and the remaining 30% as testing dataset. Besides, 10% of samples are selected as the validation dataset to overcome overfitting.

We construct an identically structured but completely independent model for each subject, and the final result is the average result of ten training sessions. The kappa score is a useful metric in multiclass problems because the correct classification and incorrect classification are considered. It can be calculated by the following formula:

(12) $$\begin{array}{c} \text{kappa}=\frac{p_{o}-p_{e}}{1-p_{e}}, \end{array}$$

where *p*_*o*_ is the average classification accuracy rate, and *p*_*e*_is the proportion of chance expected agreement. The average accuracy rate of 9 subjects is 83.38%, and the value of kappa is 0.784 for dataset I; the average accuracy of 3 subjects is 89.45%, and the value of kappa is 0.859 for dataset II; more detailed results are listed in Tables 3–6.

Further, some comparative models are also built to test the reliability of our method, which are built as follows:

*Model A*: it removed the *μ* band

*Model B*: it removed the *β* band

*Model C*: it added two convolutional layers after the second and third convolutional layers of *μ* band

*Model D*: it added two convolutional layers after the second and third convolutional layers of *β* band

*Model E*: it changed the size of the first layer of convolutional kernel to 2 × 32

**Figure 7:**
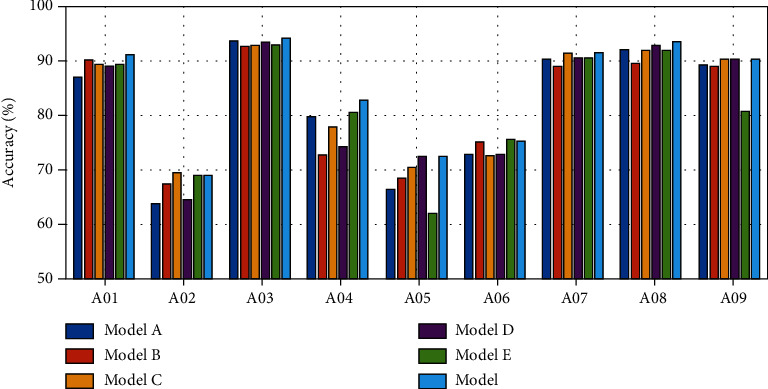
The comparison of different models.

When removing one layer of multifrequency brain network (model A and model B), a portion of the information will be eliminated, which leads to a decrease in accuracy. When the number of convolution kernels is increased in multifrequency networks (model C and model D), the average accuracy will decrease, which is due to overfitting phenomenon caused by overcomplex deep learning models. In model E, due to the reduction size the convolutional kernel into 2 × 32, only two bands of features can be fused during the convolution, which affects the performance of the entire model. More detailed results of all subjects of dataset I are listed in Figure 7.

**Figure 8:**
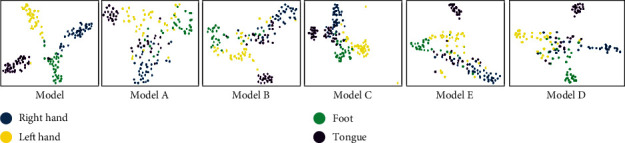
The feature map of subject A01 from dataset I.

To further prove that the proposed MFCNN model learning features are highly differentiable, we use the t-SNE algorithm to downscale the high-dimensional features learned by the MFCNN network and present them in a two-dimensional space. We show the feature map of subject A01 from dataset I as an example in Figure 8. According to the distribution of features in the figure, we clearly find that the model in Figure 8 has the best classification effect, which proves that the MFCNN model is very powerful in extracting features from the brain network and makes them inherently distinguishable.

**Figure 9:**
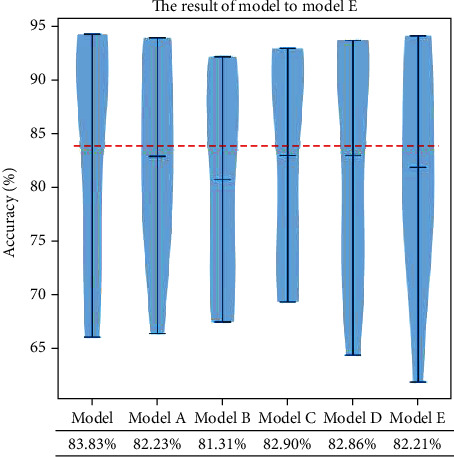
The violin plot of six models. The red dotted line indicates the highest average accuracy rate. The blue-shaded areas indicate the distribution of classification accuracy.

In order to compare the classification effects between different models visually, we plot the results of models as the violin plot in Figure 9. As shown in the figure, each model's three lines indicate the highest classification accuracy, the average classification accuracy, and the lowest classification accuracy among the nine subjects. Our model achieves the highest average accuracy, and the distribution of accuracy is more towards the top, which shows our model design is reasonable.

We select some existing works to evaluate model performance. Tables 3–6 exhibit the accuracy and kappa value of dataset I and dataset II. All these existing works have attempted a variety of feature extraction or classification methods. Our framework inherits the strengths of existing works and obtains the highest classification accuracy of all existing works. All results show that the multifrequency brain network-based deep learning framework has unique advantage in classifying MI signals.

## 5. Conclusions

MI signals have received extensive attention in the stroke rehabilitation system, and accurate decoding of MI signals plays an essential role in rehabilitating stroke patients. In this paper, we have proposed a novel deep learning framework based on multifrequency brain network, which allows decoding the multiclass motor imagery tasks accurately. The multifrequency brain network integrates time-, frequency-, and channel-related information, which can represent the brain activity during MI tasks effectively. The visualization results of the multifrequency brain network demonstrated that the multifrequency brain network can depict the brain activity during different class MI tasks dramatically. The feature matrix derived from the FBCSP algorithm can provide more precise frequency characteristics and improve the accuracy of the model. Then, we propose an MFCNN model based on the characteristics of these features; these features could be learned and integrated by the deep learning framework effectively. Our model is tested on the public BCI competition IV dataset 2a and public BCI competition III dataset IIIa, and both achieved outstanding results. Specifically, the classification accuracy of 83.83% and a kappa value of 0.784 are achieved for the first dataset, and the classification accuracy of 89.45% and a kappa value of 0.859 are achieved for the second dataset. The results indicate that our framework can give a better performance and achieve high classification accuracy compared with existing works. Considering the validity and generality of our framework, we hope that it could be applied to more neural rehabilitation fields, e.g., the rehabilitation of stroke patients, in future studies.

## Figures and Tables

**Table 1:** Deep learning network structure.

Name	Parameters	Output shape

MFCNN	Conv, 6∗6@32(elu)^1^	22, 22, 32
	Maxpooling, 2∗2	11, 11, 32
	Conv, 4∗4@64(elu)	11, 11, 64
	Conv, 3∗3@64(elu)	11, 11, 64
	Maxpooling, 2∗2	5, 5, 64
	Conv, 3∗3@64(elu)	5, 5, 64
	Flatten	1600
	Dense,64,(elu)	64

FBCSP	Conv, 3∗32@32(elu)	41, 233, 32
	Maxpooling, 2∗2	20, 116, 32
	Conv, 3∗16@64(elu)	18, 101, 64
	Conv, 2∗8@64(elu)	17, 94, 64
	Flatten	102272
	Dense,64,(elu)	64

Conv, 6∗6@32(elu) means 32 kernels with 6∗6 size and ELU activation in this convolutional layer.

**Table 2:** 11 introduced time windows.

No.	1	2	3	4	5	6	7	8	9	10	11
Start time	2.5 s	3 s	3.5 s	4 s	4.5 s	5 s	2.5 s	3 s	3.5 s	4 s	2.5 s
End time	3.5 s	4 s	4.5 s	5 s	5.5 s	6 s	4.5 s	5 s	5.5 s	6 s	6 s

**Table 3:** The accuracy of existing methods and our model (dataset I).

Subject	Methods							
	TSSM and LDA [47]	TSLDA [48]	C2CM [49]	MMISS [50]	Multiview [51]	FBN [30]	FBSF-TSCNN [35]	Our model
A01	81.80%	80.50%	87.50%	79.07%	86.60%	82.76%	85.80%	**91.26%**
A02	65.60%	51.30%	65.28%	46.38%	61.26%	65.52%	60.10%	**66.08%**
A03	88.80%	87.50%	90.28%	88.83%	87.27%	87.93%	87.80%	**94.11%**
A04	63.70%	59.30%	66.67%	62.42%	75.20%	77.59%	64.20%	**82.86%**
A05	62.90%	45.00%	62.50%	48.70%	64.55%	72.41%	48.60%	**72.53%**
A06	59.50%	55.30%	45.49%	46.38%	65.91%	70.49%	56.90%	**72.21%**
A07	86.60%	85.10%	89.58%	81.32%	83.78%	82.76%	83.00%	**91.64%**
A08	85.10%	84.80%	83.33%	80.95%	89.91%	87.93%	81.60%	**93.35%**
A09	90.00%	86.10%	79.51%	81.25%	92.08%	89.66%	85.80%	**90.40%**
Mean	76.00%	71.32%	74.46%	68.37%	78.51%	79.67%	72.00%	**83.83%**

**Table 4:** The kappa value of existing methods and our model (dataset I).

Subject	Methods							
	TSSM and LDA [47]	TSLDA [48]	C2CM [49]	MMISS [50]	Multiview [51]	FBN [30]	FBSF-TSCNN [35]	Our model
A01	0.757	0.740	0.833	0.721	0.821	0.770	0.810	**0.883**
A02	0.541	0.350	0.537	0.285	0.484	0.540	0.468	**0.547**
A03	0.850	0.833	0.870	0.851	0.830	0.839	0.838	**0.921**
A04	0.516	0.457	0.556	0.499	0.669	0.701	0.523	**0.771**
A05	0.505	0.267	0.500	0.316	0.527	0.632	0.315	**0.633**
A06	0.460	0.404	0.273	0.285	0.545	0.606	0.426	**0.629**
A07	0.821	0.801	0.861	0.751	0.784	0.770	0.773	**0.889**
A08	0.821	0.797	0.778	0.746	0.866	0.839	0.755	**0.911**
A09	0.867	0.814	0.727	0.750	0.894	0.862	0.736	**0.782**
Mean	0.680	0.618	0.659	0.578	0.713	0.729	0.627	**0.784**

**Table 5:** The accuracy of existing methods and our model (dataset II).

Subject	Methods			
	FLS [52]	STFT [53]	MFTFS [54]	Our model
k3b	**91.80%**	73.40%	79.00%	91.67%
k6b	75.60%	78.30%	84.00%	**82.25%**
l1b	92.20%	77.91%	89.00%	**94.17%**
Mean	86.50%	76.54%	84.00%	**89.45%**

**Table 6:** The kappa value of existing methods and our model (dataset II).

Subject	Methods			
	FLS [52]	STFT [53]	MFTFS [54]	Our model
k3b	**0.939**	0.645	0.833	0.889
k6b	0.675	0.710	0.787	**0.767**
l1b	0.896	0.705	0.853	**0.922**
Mean	0.837	0.687	0.824	**0.859**

## Data Availability

The dataset used in the paper is publicly available, and anyone can register and access the dataset at http://www.bbci.de/competition/iv/#dataset2a and http://www.bbci.de/competition/iii/#data_set_iiia.
